# Systemic lupus erythematosus is a risk factor for having multiple subtypes of cutaneous lupus erythematosus

**DOI:** 10.1177/09612033241311335

**Published:** 2024-12-21

**Authors:** Grace Lu, Larry Steven Brown, Benjamin F Chong

**Affiliations:** 1 Department of Dermatology, University of Texas Southwestern Medical Center, Dallas, TX, USA; 2 Department of Health Systems Research, 21114 Parkland Health, Dallas, TX, USA

**Keywords:** Cutaneous lupus erythematosus, systemic lupus erythematosus

## Abstract

**Background:**

Patients with cutaneous lupus erythematosus (CLE) can present with one or multiple different subtypes of CLE. There is limited understanding of the prevalence and associated risk factors for having multiple CLE subtype diagnoses.

**Objective:**

This study characterized the frequency and risk factors for having multiple CLE subtypes.

**Methods:**

This was a cross-sectional study of 319 patients with CLE enrolled in the University of Texas Southwestern Cutaneous Lupus Registry seen in outpatient dermatology clinics at the University of Texas Southwestern Medical Center and Parkland Health from January 1, 2009 to December 31, 2021. Demographic and clinical information was collected from each subject and compared using univariate and multivariable logistic regression analyses.

**Results:**

59 subjects (18.5%) were diagnosed with two or more CLE subtypes. Univariate analyses identified statistically significant differences in rates of systemic lupus erythematosus (SLE) diagnosis, history of positive anti-nuclear antibody, arthritis, renal disorder, and serositis in patients with multiple CLE subtype diagnoses. In the multivariable analysis, SLE diagnosis was found to be statistically significant.

**Conclusions:**

Our study showed that almost one out of five CLE patients have multiple CLE subtypes, with SLE diagnosis being a significant risk factor. Clinicians can monitor CLE patients for developing multiple subtypes and account for systemic manifestations and laboratory abnormalities associated with SLE.

## Introduction

Cutaneous lupus erythematosus (CLE) is an autoimmune skin disease that can present with or without systemic lupus erythematosus (SLE).^1^ The onset of disease typically occurs during reproductive years, between 20 and 50 years.^2^ Population studies based in the US and Europe have reported a prevalence of 70 per 100,000, with a greater prevalence in women.^2,3^ CLE can be further classified into acute, subacute, or chronic CLE subtypes.^4^ Acute CLE (ACLE) typically presents with a bilateral malar erythema and has a strong association with SLE. Subacute CLE (SCLE) manifests as annular or papulosquamous skin lesions and can be drug-induced. Chronic CLE (CCLE) has the greatest number of subtypes, with discoid lupus erythematosus (DLE) as the most common form.^1^

Prior studies of CLE patient cohorts and existing clinical trials for CLE therapies have treated subtypes of CLE as distinct entities.^5^ Among prior literature that has examined the prevalence of having multiple CLE subtypes, the patient populations have predominantly been based in Europe and Asia, with limited available demographic or clinical data for this subpopulation.^6–9^ Likewise, current CLE treatment guidelines do not differentiate between subtypes, since the existing medication regimens for CLE have primarily been adapted from SLE therapy.^10^ Currently, only hydroxychloroquine and glucocorticoids have been specifically approved by the U.S. Food and Drug Administration (FDA) for the treatment of CLE.^5,10^ Having a better understanding of the prevalence and risk factors of having multiple CLE subtypes may eventually improve therapeutic approaches and management in patients with complex CLE presentations, as first-line treatments for CLE such as anti-malarials have been shown to have varying cutaneous response rates in different CLE subtypes.^11^ Consequently, we conducted a cross-sectional study of CLE patients to assess the prevalence and risk factors of having multiple CLE subtypes. We hypothesize that a minority of CLE patients will present with multiple subtypes, with the presence of systemic lupus erythematosus (SLE) being a significant risk factor. The findings of this study will aid clinicians in monitoring for the development of multiple CLE subtypes and its associated risk factors.

## Methods

### Participants

This was a cross-sectional study of CLE patients seen at Parkland Health and University of Texas Southwestern (UTSW) Medical Center outpatient dermatology clinics between January 1, 2009 to December 31, 2021. For patients with multiple visits, the most recent clinic visit prior to December 31, 2021 was used for data analysis. Patients were required to meet CLE diagnosis via clinicopathological correlation. Patient records and photographs were reviewed to confirm the presence of specific CLE subtypes according to the Gilliam classification scheme.^4^ Exclusion criteria included patients below 18 years of age, visits outside of the time frame of interest, absence of CLE diagnosis, and unknown CLE subtype (Supplemental Figure 1). Pediatric CLE patients were excluded due to differences in clinical presentations and progression to SLE as demonstrated in prior literature, as well as limitations in obtaining informed consent from minors.^12^ Clinical data for each patient was collected by medical chart review. All patients gave informed consent, which was approved by the UTSW Institutional Review Board.

### Demographic and clinical variables

The primary outcome variable was the presence or absence of two or more CLE subtype diagnoses. The predictor variables encompassed both demographic and clinical characteristics. Demographic characteristics included race, ethnicity, age, and sex. Clinical characteristics included SLE diagnosis, smoking history, CLE disease duration, history of positive auto-antibodies, medication status, and age of CLE diagnosis. SLE diagnosis was defined according to the presence of four or more American College of Rheumatology (ACR) criteria for SLE.^13^ Patients with both malar and discoid rash, corresponding to ACLE and DLE, were reported as meeting only one ACR criteria instead of two, in order to address their greater potential for meeting SLE diagnosis. Auto-antibodies that were considered in this study include anti-nuclear antibody (ANA), anti-double-stranded DNA (anti-dsDNA), anti-SS-A, anti-SS-B, anti-Sm, and anti-RNP antibodies. Patients were classified into three medication categories, including: 1) no medications or topical medications only, 2) anti-malarials + topical medications, and 3) immunosuppressants + anti-malarials + topical medications.

### Statistical analyses

Patient characteristics were recorded as frequencies and percentages for categorical variables and means and standard deviations for continuous variables. Univariate and multivariable logistic regression analyses were performed to identify significant predictor variables associated with multiple CLE subtypes. For univariate analyses, we used Mann-Whitney U test for continuous variables and Chi-squared or Fisher’s exact tests for categorical variables. Two-sided *p* values <0.05 were considered significant. Predictors in the logistic regression model included variables significant at *p* < 0.05 from the univariate analyses and variables declared a priori (e.g. SLE diagnosis). Each variable was added to the model in stepwise selection according to these criteria. Predictor variables with a large proportion of missing values and medication status due to collinearity with SLE diagnosis were excluded from multivariable analysis. All statistical analyses were performed using MATLAB version 2022b and SPSS version 25.

## Results

**Figure 1.**
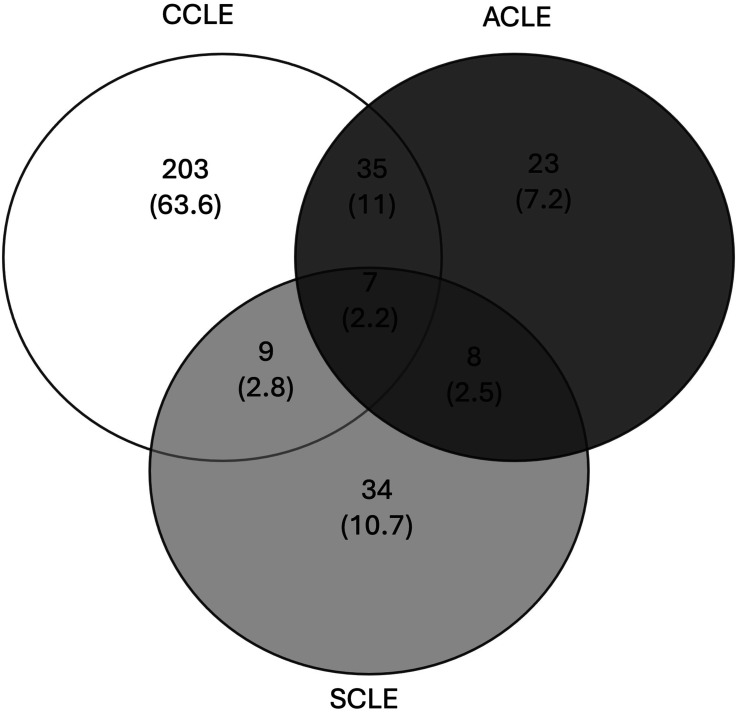
CLE subtype distribution. The Venn diagram depicts the number and percentage (noted in parenthesis) of patients with one and multiple CLE subtypes (*n* = 319). Abbreviations: ACLE: Acute Cutaneous Lupus Erythematosus; CCLE: Chronic Cutaneous Lupus Erythematosus; SCLE: Subacute Cutaneous Lupus Erythematosus.

Of the 319 patients meeting study criteria, the majority of patients were female (83.4%), and nearly half of the patients were Black Non-Hispanic (49.5%). Over half of patients were also diagnosed with SLE (53%) and on combination therapy involving immunosuppressants, anti-malarials, and/or topical medications (59.6%) (Table 1). Chronic CLE was the most common subtype diagnosis, with 79.6% of patients having this diagnosis, and 70.5% of patients being diagnosed with DLE. Fifty-nine patients (18.5%) were diagnosed with two or more CLE subtypes. The most common overlapping subtype diagnosis was ACLE and CCLE, which was found in 35 patients (11%), representing 59.3% of patients with multiple subtypes (Figure 1).

**Table 1.** Univariate analysis of demographic and clinical characteristics of patients with and without multiple CLE subtypes.

Patient characteristics	Total (*n* = 319)	1 CLE Subtype (*n* = 260)	>1 CLE Subtypes (*n* = 59)	*p* value
Age at final visit (years), median (IQR)	48.4 (36.4–57.0)	49.1 (37.9–58.8)	46.3 (31.2–53.5)	0.02^j^
Gender, n (%)				0.14^k^
Male	53 (16.6)	47 (18.1)	6 (10.2)	
Female	266 (83.4)	213 (81.9)	53 (89.8)	
Race, n (%)				0.56^l^
White Non-Hispanic	101 (31.7)	83 (31.9)	18 (30.5)	
White Hispanic	40 (12.5)	32 (12.3)	8 (13.6)	
Black^a^	158 (49.5)	131 (50.4)	27 (45.8)	
Other^b^	20 (6.3)	14 (5.4)	6 (10.2)	
Age at diagnosis (years), median (IQR)^c^	38 (28–51)	39 (30–52)	31 (23–46)	0.006^j^
Disease duration (years), median (IQR)	4.62 (1.66–12.04)	4.88 (1.47–12.09)	4.46 (2.07–11.90)	0.45^j^
Follow up duration (years), median (IQR)^d^	0.25 (0–2.26)	0.25 (0–2.19)	0.49 (0–2.68)	0.71^j^
CLE subtype, n (%)				
ACLE		23 (8.8)	50 (84.7)	
SCLE		34 (13.1)	24 (40.7)	
CCLE		203 (78.1)	51 (86.4)	
DLE		177 (68.1)	48 (81.4)	
LEP		19 (7.3)	2 (3.9)	
LET		23 (8.8	4 (6.8)	
Chilblains		7 (2.7)	1 (1.7)	
Smoking status, n (%)^e^				0.25^k^
Current	82 (27)	72 (28.9)	10 (18.2)	
Past	55 (18.1)	43 (17.3)	12 (21.8)	
Never	167 (54.9)	134 (53.8)	33 (60.0)	
Medication status, n (%)^f^				<.001^l^
None or topicals only	38 (11.9)	37 (14.2)	1 (1.7)	
Anti-malarials ± topicals	91 (28.5)	84 (32.3)	7 (11.9)	
Immunosuppressants ± anti-malarials ± topicals	190 (59.6)	139 (53.5)	51 (86.4)	
SLE diagnosis, n (%)^g^				<0.001^l^
Yes	168 (52.7)	117 (45.0)	51 (86.4)	
No	151 (47.3)	143 (55.0)	8 (13.6)	
Extracutaneous manifestations of SLE, n (%)				
Arthritis	95 (29.8)	65 (25.0)	30 (50.8)	<0.001^l^
Serositis	30 (9.4)	19 (7.3)	11 (18.6)	0.01^l^
Renal disorder	55 (17.2)	35 (13.5)	20 (33.9)	<0.001^l^
Positive ANA	232 (72.7)	179 (68.8)	53 (89.8)	<0.001^k^
Positive anti-ds-DNA^h^	94 (36.0)	57 (27.7)	37 (67.3)	<0.001^l^
Positive anti-Sm^i^	83 (33.7)	54 (27.4)	29 (59.2)	<0.001^l^

ACLE: acute cutaneous lupus erythematosus; ACR: American College of Rheumatology; ANA: anti-nuclear antibody; DLE: discoid lupus erythematosus; IQR: interquartile range; LEP: lupus erythematosus panniculitis; LET: lupus erythematosus tumidus; SLE: systemic lupus erythematosus

^a^Black consisted of 158 Black Non-Hispanic and 1 Black Hispanic patient.

^b^Other consisted of 14 Asian patients, 3 mixed race patients and 1 Middle Eastern patient, and 2 unspecified patients.

^c^Age at diagnosis was not recorded for 11 patients.

^d^167 patients had > 1 clinic visits.

^e^Smoking status was not recorded for 15 patients.

^f^3 patients were on no medications and 35 patients were on topical medications.

^g^Patients with ACLE and DLE had to meet one additional ACR criteria for SLE to establish diagnosis.

^h^anti-ds-DNA antibodies were not obtained for 58 patients.

^i^anti-Sm antibodies were not obtained for 73 patients.

^j^*p* value was calculated with Mann-Whitney U test.

^k^*p* value was calculated with χ^2^ test.

^l^*p* value was calculated with Fischer’s exact test.

### Univariate analysis

Univariate analyses were performed to identify risk factors associated with multiple CLE subtype diagnoses. Clinical risk factors associated with an increased likelihood of having multiple CLE subtypes included age at diagnosis, SLE diagnosis, medication status, history of positive auto-antibodies, and all ACR criteria excluding discoid rash (Table 1). Patients with multiple CLE subtypes (median age: 31 years, IQR: 23-46) were diagnosed at a younger age compared to patients with one CLE subtype (median age: 39 years, IQR: 30-52, *p = .006*). They were also more likely to be diagnosed with SLE (86.4% vs 45%, *p < .001*) and manifest various ACR criteria for SLE diagnosis, including arthritis (50.8% vs 25%, *p < .001*), renal disorder (33.9% vs 13.5%, *p < .001*), and serositis (18.6% vs 7.31%, *p = .01*). Likewise, they were also more likely to have a history of auto-antibodies, including ANA (89.8% vs 68.8%, *p < .001*), anti-ds-DNA (67.3% vs 27.7%, *p < .001*), and anti-Sm (59.2% vs 27.4%, *p < .001*). Lastly, patients with multiple CLE subtypes were more likely to be treated with combination therapy involving immunosuppressants, anti-malarials, and/or topicals (86.4% vs 53.5%, *p < .001*) (Table 1).

### Multivariable analysis

Predictor variables in the logistic regression model included SLE diagnosis, renal disorder, arthritis, serositis, age at diagnosis, and history of positive ANA . Of these variables, only SLE diagnosis was considered statistically significant (odds ratio: 5.09, 95% CI: 1.89-13.68, *p < .01*), suggesting that patients with multiple CLE subtypes are more likely to also be diagnosed with SLE, whereas the other variables in the model are not different between CLE subtypes (Table 2). The correlation matrix for the full model ranged from −0.44 (ANA and SLE) to 0.09 (Arthritis and Renal Disorder). The R-squared was 0.188 (goodness of fit test *p*-value = .55). The reduced model with only SLE diagnosis in the model had odds ratio: 7.74, 95% CI 3.53-16.95, *p* < .01. The R-squared value was 0.175 (goodness of fit test *p* < .01).

**Table 2.** Multivariable analysis of patient factors associated with multiple CLE subtypes.

Clinical characteristics	Odds ratio (95% CI)	*p* value
Age at diagnosis	1.00 (1.00 - 1.00)	0.82
Positive ANA	1.23 (0.43 - 3.54)	0.71
SLE diagnosis, n (%)	5.09 (1.89 – 13.68)	<0.01
Renal disorder	1.48 (0.73 – 3.00)	0.28
Arthritis	1.41 (0.72 - 2.79)	0.32
Serositis	1.16 (0.48 - 2.79)	0.74

ANA; anti-nuclear antibody; CI: confidence interval; SLE: systemic lupus erythematosus.

## Discussion

In this cross-sectional study of 319 CLE patients, we found that multiple CLE subtype diagnoses occurred in 18.5% of patients, with SLE diagnosis being a significant risk factor. The prevalence of having multiple CLE subtype diagnoses was found to be lower in our patient population compared to prior cohort studies based in Asia and Europe, which ranged from 22 to 35%.^6–9^ These differences may be due to variations in both sample sizes and clinical characteristics of the CLE patient groups, as many of these studies included patient cohorts comprised of over 1000 individuals and had higher proportions of patients diagnosed with ACLE. In contrast to previous literature, we also found that SLE diagnosis was a significant risk factor for having multiple CLE subtypes. Though some studies did demonstrate a higher prevalence of having multiple CLE subtype diagnoses in CLE patients with systemic disease, this was not found to be statistically significant.^7,9^ However, the studies that demonstrated these findings had differing groups of comparison, examining patients with isolated CLE versus CLE with laboratory SLE, as well as patients with isolated CLE, isolated SLE, or CLE with SLE.^7,9^

Although the presence of systemic symptoms and a history of positive auto-antibodies were considered statistically significant in the univariate analysis, these risk factors are accounted for in the ACR criteria for SLE diagnosis, which likely explains why these variables were ultimately not significant in the multivariable logistic regression analysis. CLE subtype diagnoses are also included in the criteria for SLE diagnosis, though this factor was adjusted by considering the presence of malar and discoid rash as meeting one ACR criteria for SLE.

Given that a sizeable proportion of CLE patients have been diagnosed with two or more subtypes, these results affirm the importance of considering complex CLE presentations in population-based studies, as well as clinical trials. it would be valuable for clinicians to monitor for the development of multiple CLE subtypes and account for potential associated risk factors, such as SLE diagnosis. Assessing patients for systemic symptoms and laboratory abnormalities (such as auto-antibodies) may be useful tools in the surveillance of developing multiple CLE subtypes. With regards to disease management, patients with multiple CLE subtypes may require multimodal treatment involving a combination of topicals, anti-malarials, and/or immunosuppressants, given its association with SLE diagnosis.

Limitations of this study include the single-center nature, which limits generalizability. The frequency of ACLE also may have been underestimated, as ACLE diagnosis was confirmed primarily through careful history taking and often not clinically observed, given the transient nature of the disease. We were also unable to follow the temporal progression of developing multiple CLE subtypes, as the majority (93.2%) of patients with multiple CLE subtypes had already been diagnosed at initial presentation. Though alternative classification criteria for SLE diagnosis have been proposed by the Systemic Lupus International Collaborating Clinics and European League Against Rheumatism/American College of Rheumatology in recent years, the 1982 ACR criteria was selected for our study to account for consistency in SLE diagnostic criteria throughout the length of follow-up, which preceded the advent of these new criteria.^14,15^

This single-center cross-sectional study found that nearly 1 in 5 patients with CLE have multiple subtype diagnoses, with SLE being a significant risk factor. Future directions include prospective studies that examine the temporal progression of multiple CLE subtypes and its associated risk factors, since the majority of patients in the UTSW Cutaneous Lupus Registry were already diagnosed with multiple subtypes at initial presentation. We also plan to examine whether having multiple subtypes affects patient outcomes, such as treatment response, quality of life, and other domains.

## Supplemental Material

Supplemental Material - Systemic lupus erythematosus is a risk factor for having multiple subtypes of cutaneous lupus erythematosus Supplemental Material for Systemic lupus erythematosus is a risk factor for having multiple subtypes of cutaneous lupus erythematosus by Grace Lu, L Steven Brown and Benjamin F Chong in Lupus

## Appendix

### Abbreviations

ACLE: Acute Cutaneous Lupus Erythematosus
ACR: American College of Rheumatology
ANA: Anti-nuclear antibody
Anti-dsDNA: Anti-double-stranded DNA
CCLE: Chronic Cutaneous Lupus Erythematosus
DLE: Discoid Lupus Erythematosus
IQR: Interquartile Range
SCLE: Subacute Cutaneous Lupus Erythematosus
SLE: Systemic Lupus Erythematosus
UTSW: University of Texas Southwestern Medical Center

## References

[1] Daniel JW . Dubois' lupus erythematosus and related syndromes. 9th ed. St Louis, MO: Elsevier, 2018.

[2] Elmgren J Nyberg F . Clinical aspects of cutaneous lupus erythematosus. Front Med 2022; 9: 984229. 20230109. DOI: 10.3389/fmed.2022.984229. PMC9868707 36698816

[3] Stull C Sprow G Werth VP . Cutaneous involvement in systemic lupus erythematosus: a review for the rheumatologist. J Rheumatol 2023; 50: 27–35. 20220915. DOI: 10.3899/jrheum.220089. PMC10152495 36109075

[4] Gilliam JN Sontheimer RD . Distinctive cutaneous subsets in the spectrum of lupus erythematosus. J Am Acad Dermatol 1981; 4: 471–475. DOI: 10.1016/s0190-9622(81)80261-7. 7229150

[5] Xie L Lopes Almeida Gomes L Stone CJ , et al. An update on clinical trials for cutaneous lupus erythematosus. J Dermatol 2024; 51: 885–894. 20240315. DOI: 10.1111/1346-8138.17161. 38491743 PMC11222050

[6] Biazar C Sigges J Patsinakidis N , et al. Cutaneous lupus erythematosus: first multicenter database analysis of 1002 patients from the European Society of Cutaneous Lupus Erythematosus (EUSCLE). Autoimmun Rev 2013; 12: 444–454. 20120918. DOI: 10.1016/j.autrev.2012.08.019. 23000206

[7] Masseran C Perray L Murat de Montai Q , et al. Comparison of patients with isolated cutaneous lupus erythematosus versus systemic lupus erythematosus with cutaneous lupus erythematosus as the sole clinical feature: a monocentric study of 149 patients. J Am Acad Dermatol 2024; 90(6): 20240201. DOI: 10.1016/j.jaad.2024.01.041. 38301924

[8] Watanabe T Tsuchida T . Classification of lupus erythematosus based upon cutaneous manifestations. Dermatological, systemic and laboratory findings in 191 patients. Dermatology 1995; 190: 277–283. DOI: 10.1159/000246716. 7655105

[9] Jin H Zhou S Yu Y , et al. Panoramic view of clinical features of lupus erythematosus: a cross-sectional multicentre study from China. Lupus Sci Med 2023; 10: e000819. DOI: 10.1136/lupus-2022-000819. 36941021 PMC10030678

[10] Verdelli A Corra A Mariotti EB , et al. An update on the management of refractory cutaneous lupus erythematosus. Front Med 2022; 9: 941003. 20220923. DOI: 10.3389/fmed.2022.941003. PMC9537468 36213629

[11] Chasset F Bouaziz JD Costedoat-Chalumeau N , et al. Efficacy and comparison of antimalarials in cutaneous lupus erythematosus subtypes: a systematic review and meta-analysis. Br J Dermatol 2017; 177: 188–196. 20170505. DOI: 10.1111/bjd.15312. 28112801

[12] Dickey BZ Holland KE Drolet BA , et al. Demographic and clinical characteristics of cutaneous lupus erythematosus at a paediatric dermatology referral centre. Br J Dermatol 2013; 169: 428–433. DOI: 10.1111/bjd.12383. 23601021 PMC4764050

[13] Tan EM Cohen AS Fries JF , et al. The 1982 revised criteria for the classification of systemic lupus erythematosus. Arthritis Rheum 1982; 25: 1271–1277. DOI: 10.1002/art.1780251101. 7138600

[14] Petri M Orbai AM Alarcon GS , et al. Derivation and validation of the Systemic Lupus International Collaborating Clinics classification criteria for systemic lupus erythematosus. Arthritis Rheum 2012; 64: 2677–2686. DOI: 10.1002/art.34473. 22553077 PMC3409311

[15] Aringer M Costenbader K Daikh D , et al. European League against rheumatism/American College of Rheumatology classification criteria for systemic lupus erythematosus. Arthritis Rheumatol 2019; 71: 1400-1412. 20190806. DOI: 10.1002/art.40930. 31385462 PMC6827566

