# Supplementary figures and images for: Systemic lupus erythematosus is a risk factor for having multiple subtypes of cutaneous lupus erythematosus
Source: Lupus. 2024 Dec 21;34(2):181–6. doi: 10.1177/09612033241311335 (PMC11780969; doi:10.1177/09612033241311335)

Supplement Figure 1

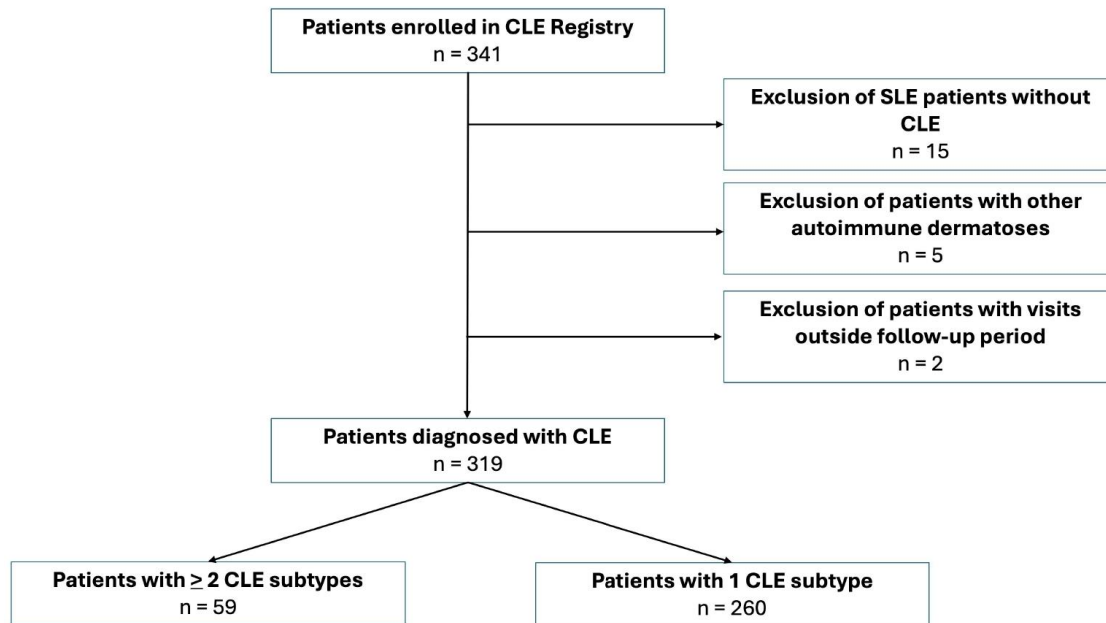

Supplement: Supplemental Material - Systemic lupus erythematosus is a risk factor for having multiple subtypes of cutaneous lupus erythematosus [file sj-pdf-1-lup-10.1177_09612033241311335.pdf]
